# Monocyte distribution width (MDW) as a new tool for the prediction of sepsis in critically ill patients: a preliminary investigation in an intensive care unit

**DOI:** 10.1186/s12873-021-00521-4

**Published:** 2021-11-22

**Authors:** Ennio Polilli, Antonella Frattari, Jessica Elisabetta Esposito, Andrea Stanziale, Giuliana Giurdanella, Giancarlo Di Iorio, Fabrizio Carinci, Giustino Parruti

**Affiliations:** 1 Clinical Pathology Unit, Pescara General Hospital, Pescara, Italy; 2 Unit of Intensive Care, Pescara General Hospital, Pescara, Italy; 3 Postgraduate School of Clinical Pathology, University of Chieti, Chieti, Italy; 4 Department of Statistical Sciences, University of Bologna, Bologna, Italy; 5 Infectious Diseases Unit, Pescara General Hospital, Pescara, Italy

**Keywords:** Sepsis, MDW, Procalcitonin, Early diagnosis, Intensive Care Unit

## Abstract

**Background:**

Monocyte Distribution Width (MDW), a simple proxy marker of innate monocyte activation, can be used for the early recognition of sepsis along with Procalcitonin. This study explored the added value of MDW as an early predictor of ensuing sepsis in patients hospitalised in an Intensive Care Unit.

**Methods:**

We performed an observational prospective monocentric study to estimate the analytical performance of MDW in detecting ensuing sepsis in a sample of consecutive patients assisted in an Intensive Care Unit for > 48 h for any reason. Demographic and clinical characteristics, past medical history and other laboratory measurements were included as potential predictors of confirmed sepsis in multivariate logistic regression.

**Results:**

A total of 211 patients were observed, 129 of whom were included in the final sample due to the suspect of ensuing sepsis; of these, 74 (57%) had a confirmed diagnosis of sepsis, which was best predicted with the combination of MDW > 23.0 and PCT > 0.5 ng/mL (Positive Predictive Value, PPV: 92.6, 95% CI: 82.1–97.9). The best MDW cut-off to rule out sepsis was ≤20.0 (Negative Predictive Value, NPV: 86.4, 95% CI: 65.1–97.1). Multivariate analyses using both MDW and PCT found a significant association for MDW > 23 only (OR:17.64, 95% CI: 5.53–67.91).

****Conclusion**:**

We found that values of MDW > 23 were associated with a high PPV for sepsis, whereas values of MDW ≤ 20 were associated with a high NPV. Our findings suggest that MDW may help clinicians to monitor ICU patients at risk of sepsis, with minimal additional efforts over standard of care.

## Background

Approximately 5 to 10% of patients with sepsis are directly hospitalized at an Intensive Care Unit (ICU) for septic shock, while 8–10% of those admitted at the ICU are at risk of developing either condition during their hospital stay [1–4]. Sepsis has been reported as a major cause of increased morbidity, length of stay and mortality among patients hospitalized in ICU for any cause [3, 5]. The risk of developing sepsis is often associated with pre-existing chronic medical conditions, e.g. diabetes, chronic inflammatory or immune disorders, obesity, cancer and heart diseases [5].

The survival of patients developing sepsis in the ICU is strictly related to an early diagnosis, as well as a prompt start of appropriate medical interventions [2, 3]. The early recognition of sepsis may be hampered by ambiguous clinical features and/or by a low sensitivity/specificity of current laboratory biomarkers. As a consequence, defining new strategies for an efficient and timely diagnosis of sepsis at admission and during the ICU stay is a well-recognised research priority [2, 6].

Several methods have been proposed for the identification of sepsis and the stratification of risk in ICU patients. Updated guidelines for sepsis management (SEPSIS-3) recommended the use of SOFA (Sequential Organ Failure Assessment) score, in order to recognise ensuing sepsis [3]. Among biomarkers of sepsis, procalcitonin (PCT) is acknowledged as the single best parameter for patients at the ICU [6–8]. Daily or twice-daily assessments recently improved sensitivity and drove therapy de-escalation [2, 9, 10]. However, even with serial PCT measurements, the level of sensitivity and specificity achieved for the prediction of sepsis was at best equal to 75% [11, 12]. Serial presepsin measurements were lately suggested to complement PCT, with additional costs [13]. Specific limitations, including the impact of renal function and immune status on physiological variations of presepsin levels, made both standardization and scaling up of presepsin quite difficult [14].

Recent findings suggested using Monocyte Distribution Width (MDW), a relatively simple proxy of innate monocyte response to bacterial or fungal bloodstream invasion, as a biomarker for the early recognition of sepsis [15].

MDW provides clinicians with essential information on the variability of cellular volume of the monocyte population in the peripheral blood. Measuring the immune state and the relevant cellular components may represent a useful research strategy towards an early recognition of sepsis and prompt monitoring of its progression. Different authors reported that changes in the morphology of monocytes and /or neutrophils reflect the state of cellular activation of innate immunity [16, 17]. At this stage, monocytes shift in the activated cellular form, moving quickly from peripheral blood to the site of infection, and change their size and volume, differentiating into amoeboid cells and increasing expression of functional markers, e.g. CD16 [18, 19]. As a consequence, the accurate measurement of monocyte size as a proxy of the degree of monocyte immune activation in septic patients may represent a viable solution to recognize the early onset of infections among patients staying longer at the ICU. Monocytes are known to increase their size upon activation in bacteriemic or fungemic patients. Therefore, measuring the spread of monocyte size in reading chambers of new generation hematologic analyzers seems like a promising strategy to monitor ensuing sepsis [15]. In a recent paper, we showed the potential benefit of MDW associated with the diagnosis of sepsis in patients admitted to an Infectious Diseases Ward [20].

In this paper, we present the results of a targeted investigation aimed at testing the use of MDW in association with serial PCT measurements, to identify a new algorithm for the early detection of sepsis in patients hospitalised at the ICU for any reason.

## Methods

### Design of the study

We performed an observational, prospective study to estimate the analytical performance of MDW in detecting sepsis or septic shock in patients hospitalized at the ICU of the General Hospital of Pescara (Abruzzo, Italy). The hospital is an urban 650-bedded tertiary facility of regional reference for adult traumas and acute diseases of neurosurgical interest. The ICU facility is a single, 11-bed unit, receiving critically ill patients from regional emergency departments, as well as from medical and surgical units in the hospital. The study was conducted in accordance with the amended Declaration of Helsinki. The local Health Administrative Board reviewed in detail the study plan prepared by the ICU and Laboratory Staff of the Pescara General Hospital. General written informed consent was available from all patients upon hospital admission, authorising the use of anonymised clinical and laboratory data for institutional research purposes. Specific informed consent was not considered required, as confidentiality was guaranteed and no specific interventions were performed beyond the ordinary good standard clinical practices (measurement of blood cell volumes and indices). Data were collected between January 1st, 2018 and December 31st, 2018 for all patients admitted to the above described ICU. To avoid inclusion of patients destined to short-term ICU stay, patients were consecutively enrolled after 48 h of hospitalization in the ICU. Demographic and clinical characteristics of patients, past medical history, and laboratory measurements were collected. During the follow-up period, enrolled patients who died from any cause were classified as non-survivors. We focused our analysis on patients with a suspicion of sepsis during stay at the ICU, comparing those with and without a confirmed diagnosis of sepsis according to the following definitions.

### Definitions

Sepsis and septic shock were diagnosed according to the diagnostic criteria of the Sepsis-3 classification (2016) [3]. Criteria for organ dysfunction were: sepsis-induced hypotension; lactate above normal upper limits; urine output < 0.5 mL/kg/h for > 2 h despite adequate fluid resuscitation or creatinine > 2.0 mg/dL (176.8 μmol/L); acute lung injury, with PaO2/inspired oxygen fraction (FiO2) < 250 mmHg in the absence of pneumonia or acute lung injury with PaO2/FiO2 < 200 mmHg in the presence of pneumonia; bilirubin > 2.0 mg/dL (34.2 μmol/L); platelet counts < 100,000/mm3. All relevant definitions were summarized in Table 1.

**Table 1:** Sepsis definition and considered laboratory parameters

		Definition	Reference
Sepsis		A life-threatening organ dysfunction caused by a dysregulated host response to infection	[3]
Organ Dysfunction		Organ dysfunction may be identified as an acute change in total SOFA score 2 points consequent to the infection	[3]
Septic Shock		Sepsis induced ipotension (Sepsis and vasopressor therapy needed to elevate MAP ≥65 mmHg and lactate > 2 mmol/L (18 mg/dL) despite adequate fluid resuscitation) or lactate above normal upper limits; urine output < 0.5 mL/kg/h for > 2 h despite adequate fluid resuscitation or creatinine > 2.0 mg/dL (176.8 μmol/L); acute lung injury, with PaO2/inspired oxygen fraction (FiO2) < 250 mmHg in the absence of pneumonia or acute lung injury with PaO2/FiO2 < 200 mmHg in the presence of pneumonia; bilirubin > 2.0 mg/dL (34.2 μmol/L); platelet counts < 100,000/mm3	[2] and [3]
Microorganism associated with resistant infections	Gram negative	-Carbapenemase-producing *Enterobacteriaceae* -Extended-spectrum β-lactamase (ESBL)-producing *Enterobacteriaceae* -Acquired AmpC β-lactamase-producing *Enterobacteriaceae* -Carbapenemase-producing *Pseudomonas* *aeruginosa* and *Acinetobacter*-group	[21, 22]
	Gram positive	-Meticillin-resistant *Staphylococcus aureus* (MRSA) -Glycopeptide non-susceptible *Staphylococcus aureus* -Vancomycin resistant *Enterococcus* -Penicillin non-susceptible *Streptococcus pneumoniae*	[21, 22]
Relevant considered laboratory parameters	MDW	Morphologic changes in the size of monocytes measured using the volume, conductivity, and scatter (VCS parameters) from the automated hematology analyzers.	[15, 20, 23]
	PCT	PCT > 0.5 ng/mL were considered the best cut off for sepsis management	[24]

### Microbiological definitions and methodology

Gram negative alert organisms considered were: meropenem/imipenem resistant *Klebsiella pneumoniae*; meropenem/imipenem resistant *Pseudomonas aeruginosa*; meropenem/imipenem resistant *Acinetobacter baumannii*. Gram positive alert organisms were methicillin resistant *Staphylococcus aureus* and methicillin resistant *Coagulase Negative Staphylococcus* [21, 22]. Identification and sensitivity assays were performed at the central Microbiology Unit, Pescara General Hospital, using the Vitek2 system (bioMérieux, France), Accelerate Pheno Test (Accelerate Diagnostics, US), GeneXpert (Cepheid, US), as well as disc diffusion methods and agar MIC determinations (antibiotic discs and MIC test strips by Liofilchem, Italy) according to the EUCAST 2017 guidelines. All methods were not modified across the study period.

### Laboratory parameters and MDW determination

Blood cell counts (Red Bood Cells, RBC; White Blood Cell Count, WBC; Hemoglobin, HGB; Hematocrit, HCT; Mean Cell Volume, MCV; Mean Cell Hemoglobin Concentration, MCHC; Mean Platelet Volume, MPV; Platelet Cell Width, PDW; Red Cell Distribution Width, RDW), platelet indices and MDW determinations were analyzed with the instrument UniCel DxH800 hematologic analyzer system (Beckman Coulter, Inc., Brea, California).

In addition to traditional numerical parameters (total number and percentages of WBCs), hematological analyses measured positional parameters also known as Cell Population Data. These represent numerical values correlated to the intrinsic biophysical properties and morphology of leukocytes. UniCel DxH 800 haematology analyser measures positional parameter with VCS technology (Volume, Conductivity, and Scatter), using 3 independent energy sources simultaneously: direct current impedance to measure cell volume of all cell types; radio frequency opacity, to characterize conductivity for internal composition of each cell; a laser beam to measure light scatter for cytoplasmic granularity and nuclear structure [20, 25, 26].

Monocyte distribution width (MDW) was measured as described in Chaves et al. (2005) [27]. Briefly, MDW is estimated in the adult population utilizing Volume, Conductivity, and Scatter (VCS) technologies. VCS parameters can detect morphologic changes in immature and reactive cells, similarly to microscopic evaluation of a peripheral blood smear. MDW was analyzed on the first blood sample at hospital entry and then every day with the daily control of Complete Blood Count (CBC); MDW values were omitted in medical reports, because they were assessed for research purposes only, and were retrieved for statistical analyses. Values of MDW were paired with PCT values for monitoring of sepsis in ICU patients. All determinations of MDW were obtained from a K3EDTA whole-blood venous sample, assayed within 2 h of collection, in the same tubes of blood used for other CBC determinations. Turnaround time for such measurements was within the time of CBC counts. Quality control was carried out by monitoring performances of diagnostic processes using commercial controls. Controls with known characteristics were analyzed daily in the same way as samples, and results of the analyzed controls were compared with standard characteristics using statistical methods calculated by the same instrument. Quality control of CBC and cell population data including MDW were performed every 24 h before analytic session with both COULTER LATRON CP-X Control and COULTER® 6C Cell Control. They were utilized also every after of an unexpected injury of the instrument. COULTER LATRON CP-X Control is a suspension of stable polystyrene particles of uniform size with a diameter CV ≤3.0%. It is used to monitor the stability of the electrical processing and the fluidic flow rate systems used to measure the volume, conductivity and light scattering characteristics of cells as they pass through the flow cell. COULTER® 6C Cell is performed by 3 control samples that is utilized to monitoring of system performance for all directly measured and is utilized to calculated also positional parameters. Acceptability of control values take place automatically through dedicated software of the same analyzer hematological instrument. Producers of haematological analyzers do not provide any unit for MDW and other positional parameters, as previously published [20, 28]. Positional parameters derive from an algorithmic application that transforms femtoliters in positions on the x-axis of the scatterplot, of entity proportionate to the value of the cell volume [20].

### Statistical analysis

All results were analysed using the confirmed status of sepsis as the main outcome of interest. The following demographic and clinical factors were used for descriptive analysis: age, gender, presence of comorbidity, SOFA score, SAPS II (Simplified Acute Physiology Score) score, previous antibiotic exposure (defined as treatment for at least one week with either quinolone, beta-lactam, or carbapenem prescribed in the month preceding hospitalization), previous surgery (defined as surgery in the month preceding ICU admission or during ICU stay), blood counts, other available biomarkers of sepsis (PCT in nearly all cases) and microbiological isolates, including Multi Drug Resistant Organisms, MDRO(s).

Significance levels and 95% confidence intervals were based on an alpha = .05. The differences between patients with or without confirmed sepsis were tested using chi-square for categorical variables, while Student’s t-test and non-parametric Kruskal–Wallis rank tests were used for normal and non-normal continuous variables respectively [29].

The reliability of MDW and PCT in predicting sepsis was investigated through ROC analysis of varying thresholds, including the calculation of Sensitivity, Specificity, Positive Predictive Value (PPV), Negative Predictive Value (NPV) and the Area Under the Curve (AUC) [30]. Optimal thresholds for MDW and PCT were defined as those achieving the maximum value of Youden index. Cut-offs for ROC analysis were defined based upon the results obtained from the maximum level above, as well as from previous literature, including a value of 20.0 for high NPV to rule out sepsis [15, 20] and 22.0 as the best statistical cut-off for the prediction of sepsis [20]. ROC curves were produced along with their 95% confidence bands, using all possible cut-offs of MDW and PCT. The De Long’s test for two correlated ROC curves was used to test the significance of the difference in terms of AUC.

The level of association between different combinations of optimal thresholds for MDW, PCT and a confirmed status of sepsis was measured using multivariate odds ratios with 95% confidence intervals, adjusting for all relevant demographic and clinical characteristics available through multivariate logistic regression [31].

All analyses were performed using the R statistical language [32].

## Results

**Fig. 1:**
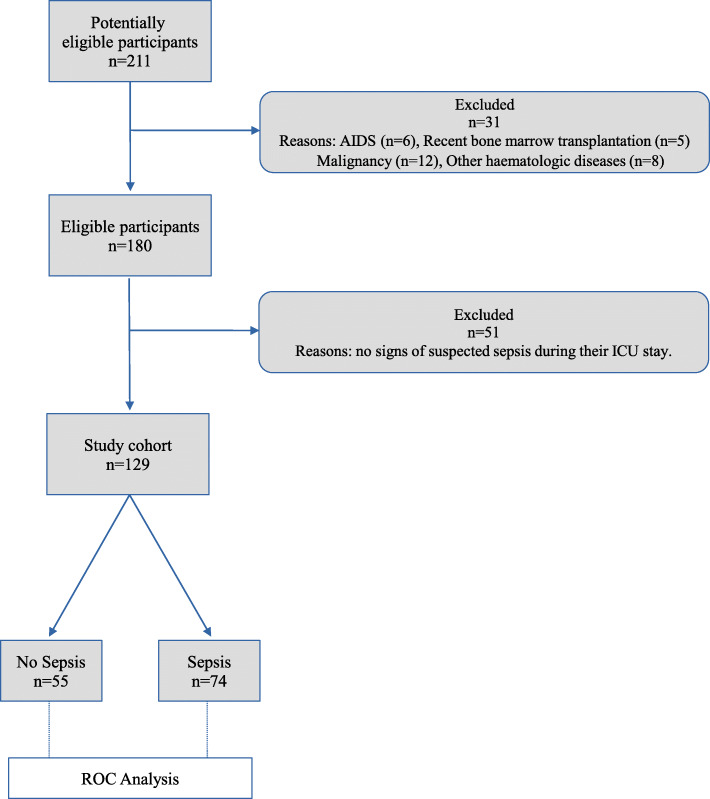
Flow chart of the selection of the study cohort for ROC Analysis

The total population of consecutive patients entering the ICU in the reference period included *N* = 211 patients. A flow chart of the selection of the study cohort is included in Fig. 1; *N* = 31 (14.6%) patients were excluded because of underlying conditions potentially associated with immune dysregulation, as AIDS (*N* = 6), recent bone marrow transplantation (*N* = 5), malignancy (*N* = 12) and other haematologic diseases (*N* = 8), all possibly influencing monocyte size and activation in response to infection [15]. The main diagnoses for *N* = 180 patients included in the study cohort are shown in Table 2. The most frequent causes of admission in ICU were intracerebral haemorrhage (17.8%), cardiovascular failure (14.4%); polytrauma (12.8%); respiratory failure (11.1%) and stroke (8.9%). A total of *N* = 51 patients were excluded from the target study group as they did not show signs of suspected sepsis during their ICU stay.

**Table 2:** Demographics, clinical characteristics and laboratory parameters of the target sample

Variables	*Study Cohort*	Target sample			
		Overall	Sepsis		
			No	Yes	*p*
N (%)	180 (100)	129 (100.0)	55 (43.0)	74 (57.0)	
*Categorical [N (%)]*					
Male Gender	107 (59)	82 (63.6)	32 (58.0)	50 (68.0)	0.362
Septic shock	21 (12)	21 (16.3)	–	21 (28.4)	–
Bacteremia	58 (32.2)	58 (45.0)	5 (9.0)	53 (72.0)	< 0.001
Mortality	46 (25)	40 (31.0)	11 (20.0)	29 (39.2)	0.03
*Continuous Normal [mean (SD)]*					
Age, years	63.7 (16.5)	62.40 (16.61)	63.11 (17.40)	61.86 (16.06)	0.680
CCI	3.5 (2.3)	3.41 (2.40)	3.40 (2.50)	3.42 (2.30)	0.960
SAPS II^†^	48.13 (15.1)	49.09 (15.06)	49.13 (13.70)	49.07 (16.07)	0.980
SOFA^†^	6.8 (3.4)	7.23 (3.51)	6.22 (2.95)	7.99 (3.71)	0.003
SOFA^††^		7.63 (3.52)	6.13 (2.69)	8.06 (3.63)	0.032
Length of stay at ICU (days)	11.56 (8.26)	12.98 (8.87)	11.49 (6.96)	14.09 (9.96)	0.083
*Continuous Non Normal [median (IQR)]*					
MDW		23.00 (21.00–27.00)	21.00 (20.00–22.30)	25.60 (23.10–29.00)	< 0.001
PCT, ng/mL		0.95 (0.19–9.8)	0.21 (0.12–0.91)	4.15 (0.60–27.00)	< 0.001
CRP, mg/L		118.96 (55.28–190.70)	108.60 (41.64–159.6)	123.73 (60.57–218.13)	0.070
WBCx10^3^/μL		10.80 (8.00–15.40)	10.35 (8.17–14.35)	10.90 (7.80–15.80)	0.950
*Categorical [N (%)]*					
Main diagnoses at ICU admission					
Intracerebral haemorrhage	32 (17.8)	24 (18.6)	14 (25.5)	10 (13.5)	0.135
Cardiovascular failure	26 (14.4)	19 (14.7)	10 (18.2)	9 (12.2)	0.482
Polytrauma	23 (12.8)	19 (14.7)	9 (16.4)	10 (13.5)	0.841
Respiratory failure	20 (11.1)	11 (8.5)	3 (5.5)	8 (10.8)	0.448
Acute ischemic Stroke	16 (8.9)	12 (9.3)	8 (14.5)	4 (5.4)	0.144
Acute kidney failure	10 (5.6)	5 (3.9)	1 (1.8)	4 (5.4)	0.560
Head trauma	9 (5.0)	6 (4.7)	3 (5.5)	3 (4.1)	1.0
Brain surgery	9 (5.0)	5 (3.9)	3 (5.5)	2 (2.7)	0.734
Acidosis in metformin use	5 (2.8)	3 (2.3)	1 (1.8)	2 (2.7)	1.0
Septic shock	4 (2.2)	4 (3.1)	0 (0.0)	4 (5.4)	0.215
Haemorrhagic shock	4 (2.2)	3 (2.3)	0 (0.0)	3 (4.1)	0.357
Peritonitis	3 (1.7)	3 (2.3)	0 (0.0)	3 (4.1)	0.357
Acute pancreatitis	2 (1.1)	2 (1.6)	0 (0.0)	2 (2.7)	0.611
Coma in encephalitis	2 (1.1)	2 (1.6)	0 (0.0)	2 (2.7)	0.611
Consequence of Duodeno-cephalo-Pancreatectomy	1 (0.6)	1 (0.8)	0 (0.0)	1 (1.4)	1.0
Anaphylactic shock	1 (0.6)	1 (0.8)	0 (0.0)	1 (1.4)	1.0
Other	13 (7.2)	9 (7.0)	3 (5.5)	6 (8.1)	0.813

*CCI* Charlson Comorbidity Index, *SAPS* Simplified Acute Physiology Score, *SOFA* Sequential Organ Failure Assessment, *PCT* Procalcitonin; CRP: C-Reactive Protein; *MDW* Monocyte Distribution Width, *WBC* White Blood Cell Count; ^†^at ICU admission; ^††^at sepsis diagnosis, available for 58 patients

Alongside *N* = 4 patients with septic shock at ICU presentation, additional *N* = 125 patients presented a suspicion of sepsis during hospitalization. Therefore, *N* = 129 patients, forming the target sample for the present investigation, underwent blood cultures and other microbiological and biochemical assays. Their baseline characteristics were in fair overlap with those of the whole study cohort.

Demographic and clinical characteristics of patients included in the target sample are reported along with laboratory parameters in Table 2.

Significant differences between patients with and without sepsis were found for mortality rates (40.5% vs 20.0%, *p* = 0.01), average SOFA score at entry (7.99 vs 6.22, *p* = 0.004), median values of PCT (4.15 ng/mL vs 0.21 ng/mL, *p* < 0.001) and MDW (25.6 vs 21.0, *p* < 0.001). No association was found for age, male gender, Charlson Comorbidity Index (CCI), WBC, C-Reactive Protein (CRP) and length of stay (Table 2).

**Fig. 2:**
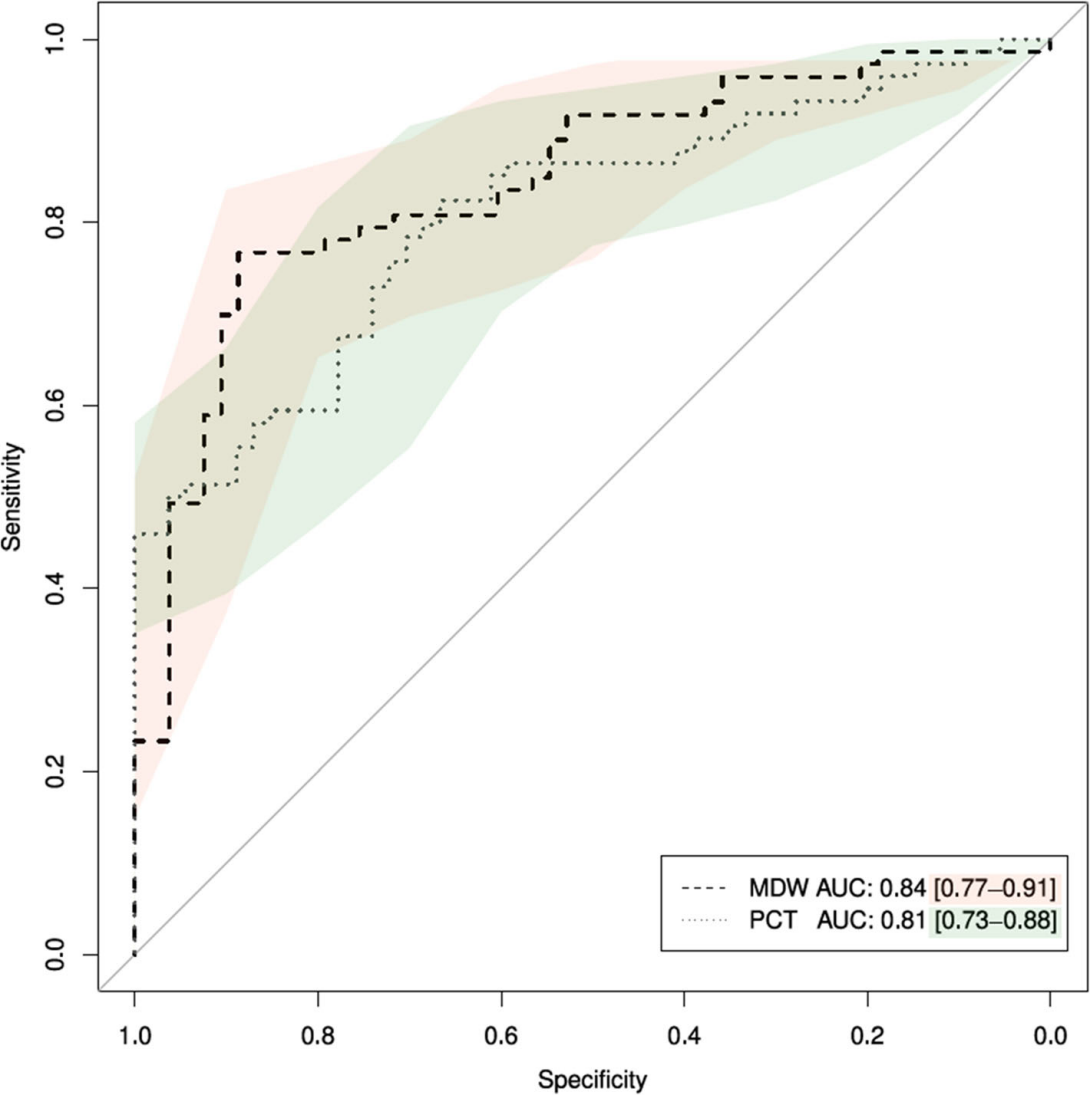
ROC curves comparing the overall prediction levels of different combinations of MDW, PCT

The summary ROC curves based upon all possible cut-offs of MDW, PCT, along with their superimposed confidence intervals, are shown in Fig. 2. The values of AUC achieved for both parameters showed to be rather comparable, with MDW achieving AUC = 0.84 (95% CI: 0.77–0.91) slightly above PCT, at an overall value of AUC = 0.81 (95% CI: 0.73–0.88). The De Long test did not reject the hypothesis of different AUC (Z = 1.0296, *p* = 0.30).

The levels of accuracy achieved with the use of alternative thresholds for MDW and PCT alone and combined are shown in Table 3. Optimal thresholds according to the Youden index were found for MDW = 23.0 and PCT = 0.5 ng/mL, respectively. The sensitivity was highest at MDW > 20.0 (95.9; 95% CI: 88.5–99.1), while specificity was highest for the composite condition of MDW > 23.0 and PCT > 0.5 ng/mL (92.5; 95% CI: 81.8–97.9).

**Table 3:** Results of ROC analysis of dichotomized values in the prediction of sepsis

Test predictors	N (%)	pre-test probability	Sensitivity (95% CI)	Specificity (95% CI)	PPV (95% CI)	NPV (95% CI)	AUC (95% CI)
MDW > 20.0	126 (97.7)	0.58	95.9 (88.5–99.1)	35.8 (23.1–50.2)	67.3 (57.4–76.2)	86.4 (65.1–97.1)	0.66 (0.59–0.73)
MDW > 22.0	126 (97.7)	0.58	79.5 (68.4–88.0)	71.7 (57.7–83.2)	79.5 (68.4–88.0)	71.7 (57.7–83.2)	0.76 (0.68–0.83)
MDW > 23.0	126 (97.7)	0.58	75.3 (63.9–84.7)	88.7 (77.0–95.7)	90.2 (79.8–96.3)	72.3 (59.8–82.7)	0.82 (0.75–0.89)
PCT > 1^a^	128 (99.2)	0.58	64.9 (52.9–75.6)	77.8 (64.4–88.0)	80.0 (67.7–89.2)	61.8 (49.2–73.3)	0.71 (0.63–0.79)
PCT > 0.5^a^	128 (99.2)	0.58	77.0 (65.8–86.0)	70.4 (56.4–82.0)	78.1 (66.9–86.9)	69.1 (55.2–80.9)	0.74 (0.66–0.82)
MDW > 20.0 and PCT > 0.5^a^	126 (97.7)	0.58	74.0 (62.4–83.5)	77.4 (63.8–87.7)	81.8 (70.4–90.2)	68.3 (55.0–79.7)	0.76 (0.68–0.83)
MDW > 22.0 and PCT > 0.5^a^	126 (97.7)	0.58	71.2 (59.4–81.2)	88.7 (77.0–95.7)	89.7 (78.8–96.1)	69.1 (56.7–79.8)	0.80 (0.73–0.87)
MDW > 23.0 and PCT > 0.5^a^	126 (97.7)	0.58	68.5 (56.6–78.9)	92.5 (81.8–97.9)	92.6 (82.1–97.9)	68.1 (56.0–78.6)	0.80 (0.74–0.87)

*PCT* Procalcitonin, *MDW* Monocyte Distribution Width, *PPV* Positive Predictive Value, *NPV* Negative Predictive Value, ^a^unit of measurement: ng/mL

The best condition to rule in sepsis was MDW > 23.0 and PCT > 0.5 ng/mL (PPV: 92.6, 95% CI: 82.1–97.9), while the best condition to rule out sepsis was MDW ≤ 20.0 (NPV: 86.4, 95% CI: 65.1–97.1). In terms of AUC, high values were found for MDW > 23.0 (0.82; 95% CI: 0.75–0.89), MDW > 22.0 and PCT > 0.5 ng/mL (0.80; 95% CI: 0.73–0.87) and MDW > 23.0 and PCT > 0.5 ng/mL (0.80; 95% CI: 0.74–0.87). Further analyses using a cutoff of PCT > 1.0 ng/mL in combination with MDW did not show any improvement in the target diagnostic parameters (results not shown).

The results of multivariate logistic regression including age, male gender, CCI, SAPS II, SOFA as adjustment terms are shown in Table 4. All terms were included in the model even if not statistically significant, as they were considered potential confounders of the association between different combinations of MDW and PCT and the diagnosis of sepsis. Several combinations of thresholds of MDW and PCT were found to be statistically significant. In particular, Model 1 found a significant association for MDW > 23 (OR: 22.65, 95% CI: 8.28–73.70), while Model 2 found a significant association for PCT > 0.5 ng/mL (OR: 7.26, 95% CI 3.05–18.46). When using both terms in the multivariate logistic regression, Model 3 highlighted a significant association only for MDW > 23 (OR:17.64, 95% CI: 5.53–67.91).

**Table 4:** Results of Logistic regression models to predict the status of confirmed sepsis in the sample

	Model 1			Model 2	Model 3	
Variables	OR (95% CI)	p	OR (95% CI)	p	OR (95% CI)	p
Age	1.00 (0.95–1.04)	0.8459	1.02 (0.98–1.07)	0.2944	1.00 (0.95–1.05)	0.9284
Male gender	2.30 (0.78–7.36)	0.1397	1.62 (0.66–4.10)	0.2952	2.30 (0.78–7.44)	0.1419
CCI	1.00 (0.73–1.38)	0.9899	0.85 (0.62–1.15)	0.2833	0.97 (0.69–1.35)	0.8366
SAPS II	0.98 (0.93–1.03)	0.4172	0.97 (0.93–1.01)	0.0924	0.98 (0.93–1.03)	0.4042
SOFA at entry	1.18 (0.98–1.46)	0.0963	1.24 (0.04–1.51)	0.0196	1.17 (0.96–1.45)	0.1337
MDW > 23	**22.65 (8.28–73.70)**	**0.0000**	–	–	**17.64 (5.53–67.91)**	**0.0000**
PCT > 0.5 ng/mL	–	–	**7.26 (3.05–18.46)**	**0.0000**	1.58 (0.46–5.09)	0.4527

We also calculated sensitivity, specificity, PPV and NPV of different MDW cut offs for the prediction of positive blood cultures (see Table 5). Best sensitivity and specificity were obtained for MDW values > 20 and > 23, respectively. The highest values of AUC were found for MDW > 22.0 (0.72; 95% CI: 0.64–0.79). Noticeably, the NPV for MDW > 20 achieved 100%, as this category included all bacteremic patients.

**Table 5:** Results of ROC analysis of values of MDW in the prediction of positive blood cultures

Variables	N	Sensitivity (95% CI)	Specificity (95% CI)	PPV (95% CI)	NPV (95% CI)	AUC (95% CI)
MDW > 20	126	100.0 (93.6–100.0)	31.4 (20.9–43.6)	53.8 (43.8–63.7)	100.0 (84.6–100.0)	0.66 (0.60–0.71)
MDW > 21	126	92.9 (82.7–98.0)	44.3 (32.4–56.7)	57.1 (46.3–67.5)	88.6 (73.3–96.8)	0.69 (0.62–0.75)
MDW > 22	126	82.1 (69.6–91.1)	61.4 (49.0–72.8)	63.0 (50.9–74.0)	81.1 (68.0–90.6)	0.72 (0.64–0.79)
MDW > 23	126	71.4 (57.8–82.7)	70.0 (57.9–80.4)	65.6 (52.3–77.3)	75.4 (63.1–85.2)	0.71 (0.63–0.79)

## Discussion

Strengthening the management of sepsis in critically ill patients can be paramount to improve quality of care and outcomes at the ICU [2, 3]. In this study, we provided new avenues to support the routine use of MDW for the early identification of sepsis among patients admitted at the ICU.

Our results are consistent with the preliminary evidence reported by relevant investigations. Recent studies showed that changes in volume of monocytes may be due to the monocyte activation upon bloodstream infections as part of innate immunity response [15, 20, 26]. To date, three large clinical studies investigated the use of MDW to predict sepsis, but they enrolled only patients admitted to Emergency Departments [15, 33, 34]. Crouser et al. showed that MDW can discriminate sepsis from SIRS and that the magnitude of MDW elevations correlate with infection severity and organ dysfunction, with MDW values rising in parallel with the severity of ensuing sepsis [15]. Further, Crouser et al. (2020) demonstrated that including MDW in the initial evaluation of patients admitted to the Emergency Department may increase the odds of early sepsis detection 6-fold using Sepsis-2 and 4-fold using Sepsis-3 assessments [33]. An additional prospective study described for the first time the ability of MDW to predict sepsis in a cohort of 260 consecutive patients hospitalized in an Infectious Diseases ward [20]. The study showed that the levels of AUC achieved by MDW and PCT for the prediction of sepsis were comparable.

The present investigation focused on results obtained during hospital stay at the ICU. In this setting, it is well known that body temperature, heart rate, organ function and tissue perfusion, are frequently abnormal for reasons that could be different from sepsis. Patients admitted at the ICU may be affected by systemic inflammation and bacterial colonization at a single or even multiple sites independently from sepsis [3, 22, 35]. Under such circumstances, the timeliness of intervention with empirical antimicrobials can be critical, as it relies upon the availability of results of microbiological assays, which may take hours to days to be delivered under standard protocols. These gaps may be life threatening in unstable patients [3, 36]. Inflammatory biomarkers measured on a daily basis during the stay at the ICU may be potentially useful to pick up early ensuing sepsis [8, 37, 38].

Previous results confirmed the role of PCT as the best biochemical marker for therapy de-escalation and outcome prediction. However, its ability to detect sepsis has been debated, considering that the maximum PPV recorded was limited to 75% [4, 39–45].

Several recent studies indicated that this novel parameter can be used to assist diagnosis in the Emergency Department, in association with SEPSIS 2 and SEPSIS 3 criteria. The first analysis of MDW in ICU was published in 2020 by Agnello et al. [46]. The authors found that patients with sepsis at entry had higher values of MDW, compared with patients without sepsis. Patients developing sepsis during their hospital stay showed higher values of MDW at the onset. Sepsis was defined by the WHO as the final stage experienced by patients with severe infectious diseases prior to death [47]. The estimated risk of death ranged between 26.7% at a general hospital and 42.6% in ICU settings (WHO), with approximately 24.4% of cases acquiring infection during their hospital stay. At the ICU, the early administration of antibiotics and a bundle of evidence-based treatments are mandatory to decrease infection -related mortality [47]. Therefore, the timeliness of diagnosis remains the most important challenge in the management of sepsis at the ICU. For this reason, Piva et al. (2021) enrolled 556 patients to evaluate the diagnostic accuracy of MDW among ICU patients. They demonstrated that MDW, measured at the onset of symptoms, has an AUC comparable with that of PCT and higher than CRP and WBC [48]. In our study, we found that using MDW alone with a cut-off of 20.0 achieved the highest levels of sensitivity (95.9%) and NPV (86.4%). These parameters indicate that MDW can be used to rule out sepsis when the patient does not exceed a level of 20.0.4 In addition, we also demonstrated that all patients with MDW ≤ 20 had invariably negative blood cultures, with a NPV of 100% for bacteremia. This procedure can be extremely convenient, as MDW can be measured routinely through a haematological analyser that is normally available to provide results for blood cell counts that can be delivered within minutes during ICU stay. These findings suggest that MDW can significantly help identifying sepsis among critically ill patients in clinical practice, with further scope for repeated measurement according to clinical judgment.

The use of PCT alone showed to be unable to achieve comparable results in terms of diagnostic performance of detecting sepsis, either with 0.5 or 1.0 ng/mL as a chosen cutoff. Nevertheless, with a limit posed at PCT > 0.5 ng/mL, the combination with different levels of MDW showed to increase specificity, at the cost of lowered sensitivity. This could be helpful to identify patients who are most likely experiencing sepsis (rule in). We found that a combination of MDW > 23 and PCT > 0.5 ng/mL not only achieved the highest specificity (92.6%), but also the highest Youden Index (0.61) and PPV (92.6%). This suggests that using both parameters is an optimal rule to identify patients with sepsis at the ICU. The choice of PCT > 0.5 ng/mL as the best cut-off is not only consistent with the available literature, but also motivated by the lack of additional gain offered by the higher level of PCT > 1.0 ng/mL.

In summary, our study identified a new candidate algorithm for the prediction of sepsis using different levels of MDW and PCT, through a balanced assessment of different diagnostic parameters. As expected, there was no single best value for sensitivity and specificity and PPV was generally higher than NPV, which may be well explained by the 0.58 pre-test probability of sepsis found in our sample. Nevertheless, based on the levels of the Youden Index and AUC, we may suggest using the following algorithm:

- check the level of MDW first: if MDW ≤ 20 rule out sepsis (NPV = 86.4);
- if MDW > 20 and MDW ≤ 23 then check PCT: if PCT > 0.5 ng/mL then if MDW ≤ 22 assign a moderate risk of sepsis (PPV = 81.8), if MDW > 22 and MDW ≤ 23 assign a high risk of sepsis (PPV = 89.7);
- if MDW > 23 assign a very high risk of sepsis (PPV = 90.2) and check PCT: if PCT > 0.5 ng/mL rule in sepsis (PPV = 92.6).

Further prospective studies are necessary to confirm the results of our exploratory investigation, indicating the role MDW as a valid diagnostic tool for the identification of sepsis, with more precise estimates obtained from a larger population.

Finally, several limitations of our study design should be considered.

Firstly, despite being based on current Sepsis-3 standards, the diagnosis of sepsis may have been potentially misclassified in some cases. Nevertheless, this problem should be limited and not affecting the statistical significance of our results.

Secondly, the study involved a limited number of patients enrolled at a single ICU, which may cast issues on the generalisability of our results to different clinical practices. However, the demographic, clinical and laboratory characteristics characterising our study population should be comparable to those found in most western countries [49].

Thirdly, specific exclusion criteria applied in our study should be taken into account when interpreting the results. In particular, the immune status of excluded patients e.g. acquired immunodeficiency, long term steroid or other immune suppressive treatments, may be associated with different monocyte volumetric variations upon bloodstream infections. Therefore, the lack of these characteristics in our study population may limit the overall generalisability of our results.

## Conclusion

Our findings suggest that MDW can be used as a novel sustainable biomarker of ensuing sepsis at the ICU, alone or in combination with PCT. Values of MDW ≤ 20 can be used to rule out sepsis (sensitivity = 95.9%, NPV = 86.4%). On the other hand, MDW > 23 can be used to rule in sepsis (PPV = 90.2%), with a slight gain when used in combination with PCT > 0.5 ng/mL (PPV = 92.6%). Further prospective and multicentric investigations are needed to validate the role of MDW, testing the same hypothesis in broader populations and different practices, possibly within a wider array of inflammation parameters and biomarkers.

## Data Availability

The datasets analysed during the current study are available from the corresponding author on reasonable request.

## Abbreviations

CCI: Charlson Comorbidity Index
SAPS: Simplified Acute Physiology Score
SOFA: Sequential Organ Failure Assessment
PCT: Procalcitonin
CRP: C-Reactive Protein
MDW: Monocyte Distribution Width
ICU: Intensive Care Unit
CBC: Complete Blood Count
PPV: Positive Predictive Value
NPV: Negative Predictive Value
